# Epithelial Intermediate Filaments: Guardians against Microbial Infection?

**DOI:** 10.3390/cells5030029

**Published:** 2016-06-27

**Authors:** Florian Geisler, Rudolf E. Leube

**Affiliations:** Institute of Molecular and Cellular Anatomy, RWTH Aachen University, Wendlingweg 2, 52074 Aachen, Germany

**Keywords:** epithelium, keratin, barrier, pathogen, virus, bacterium, parasite, *Caenorhabditis elegans*

## Abstract

Intermediate filaments are abundant cytoskeletal components of epithelial tissues. They have been implicated in overall stress protection. A hitherto poorly investigated area of research is the function of intermediate filaments as a barrier to microbial infection. This review summarizes the accumulating knowledge about this interaction. It first emphasizes the unique spatial organization of the keratin intermediate filament cytoskeleton in different epithelial tissues to protect the organism against microbial insults. We then present examples of direct interaction between viral, bacterial, and parasitic proteins and the intermediate filament system and describe how this affects the microbe-host interaction by modulating the epithelial cytoskeleton, the progression of infection, and host response. These observations not only provide novel insights into the dynamics and function of intermediate filaments but also indicate future avenues to combat microbial infection.

## 1. Intermediate Filaments: Organization and Function

Intermediate filaments (IFs) together with actin filaments and microtubules are major components of the cytoskeleton. They provide mechanical tissue stability and contribute to many cellular processes such as vesicle trafficking, organelle positioning, cell cycle regulation, differentiation, and cell motility [1,2,3,4]. Although IFs are not essential for any of these functions, they provide an abundant buffering system protecting against various types of stress, be it physical, chemical, or microbial. This property is most relevant in epithelia, which are exposed to multiple environmental stressors. 

**Figure 1:**
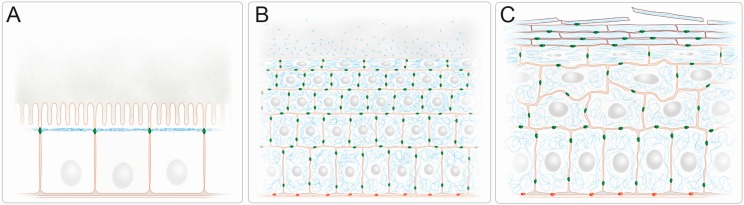
IFs form complex three-dimensional networks with cell type specific subcellular arrangements providing barrier function in simple and stratified epithelia. (**A**) Keratin IFs (blue) are subapically enriched in a dense filamentous network in the simple epithelium of the intestine. They localize just below the microvillar brush border that protrudes into the nutrient-filled intestinal lumen. The cylindrical epithelial cells are connected by junctional complexes, which encompass keratin-anchoring desmosomes (green), and rest all on a basal lamina; (**B**) The keratin IFs (blue) of the stratified epithelium of the cornea form dense 3D-networks that traverse the entire cytoplasm and are attached to desmosomes (green) at cell-cell contact sites. Keratin fragments with antibacterial activity are released into the tear fluid. The keratin cytoskeleton of the basal cells is anchored to hemidesmosomes (red), which attach to the underlying extracellular matrix of the basement membrane; (**C**) The keratin IF cytoskeleton of the epidermis, which is the prototype of a multilayered cornified epithelium, increases in density in the flattened suprabasal cell layers and becomes compacted as part of the cornified envelope of the dead cells in the uppermost *stratum corneum* which are continuously shed from the epithelium. While desmosomes (green) are present in all cell layers, hemidesmosomes (red) are restricted to the cuboidal basal cell layer.

The cytoplasmic IF cytoskeleton of mammalian epithelial cells consists of equal amounts of type I and type II keratin polypeptides. Type I and type II keratins form stable heterodimers that are arranged in parallel and are tightly attached through hydrophobic coiled-coil interactions between their α-helical central rod domains, which are flanked by variable amino- and carboxyterminal end domains [5,6,7,8]. The mechanisms of subsequent tetramer assembly and integration into mature IFs are only partly understood. In vitro observations revealed that they involve certain intermediate steps starting with two dimers associating in an antiparallel and partially staggered fashion to form the symmetric non-polar tetramer, which constitutes the main soluble keratin pool in living cells [5,9]. Tetramers associate laterally into unit length filaments that assemble longitudinally into the 8–12 nm keratin filaments [10]. These filaments form complex three-dimensional networks in vivo with cell type-specific subcellular arrangements such as the subapical enrichment in the polarized epithelial cells of the intestine, the predominant localization underneath the cell cortex in glandular epithelia and the pancytoplasmic accumulation of dense bundles in epidermal keratinocytes ([11,12]; see also Figure 1). A major tenet of this review is that this cell type-specific arrangement determines epithelial resilience against environmental insults. This notion is supported by multiple studies in cell culture systems and transgenic animals demonstrating that the presence and spatial organization of keratin IFs is a crucial prerequisite to protect epithelial cells against different kinds of mechanical and non-mechanical stress [1,13,14,15,16,17,18,19]. Furthermore, multiple human diseases attest to the important function of keratins in maintaining epithelial tissue integrity [20,21]. For example, mutations of the epidermal keratins K5 and K14 have been identified in the human skin disease *Epidermolysis bullosa simplex*, which is characterized by excessive blister formation upon minor mechanical trauma [22,23]. This goes along with the formation of large cytoplasmic aggregates containing hyperphosphorylated keratins [24,25,26]. Furthermore, it has been suggested that keratin polymorphisms render epithelial tissues more susceptible to environmental stressors [3,27,28].

This review extends previous reviews on related topics (e.g., [29,30,31]) and reviews dealing with interactions of other types of IFs with microbes (e.g., [32]). In this review, we will elaborate on the overall barrier function of keratins in stratified and simple epithelia and how this relates to specific interactions with microbial pathogens. 

## 2. Barrier Function of Intermediate Filaments in Stratified Epithelia

Epithelial cell fragility and lysis as a consequence of compromised mechanical stability are observed in a large number of epidermal keratinopathies that are caused by single point mutations in keratin-encoding genes [33,34,35]. The histological phenotypes include blister formation and hyperkeratosis [33,36,37,38,39]. Corresponding phenotypes were also described in keratin-mutant mice [40]. Consequently, dramatically increased transepidermal water loss and increased toluidine blue permeability were reported in keratin-deficient epidermis [41,42].

Besides providing a mechanical barrier, keratins have been shown to actively contribute to barrier formation in the epidermis. Thus, keratin K10-deficient mice present reduced sphingomyelinase activity, which generates ceramides that are a major component of the extracellular lipid lamellae in the epidermal *stratum corneum* [41]. Furthermore, complete absence of keratins perturbs the formation of the cornified envelope in suprabasal keratinocytes [42]. In addition, keratin depletion and presence of mutant keratins lead to reduction in junctional proteins affecting junction formation and dynamics [19,43,44,45].

Interestingly, gene expression signatures in human patients with the skin disease *Pachyonychia congenita* carrying mutations in K16 or its partner K6 as well as K16 null mice reveal an enrichment of genes involved in inflammation and innate immunity which may be a consequence of the perturbed skin barrier [46]. Furthermore, deletion of the suprabasal keratin K1 in transgenic mice resulted in inflammasome activation and IL-18 processing [47]. The observed skin pathology presenting erosions, hyperkeratosis, and barrier defects could be partially rescued by IL-18 depletion in these mice [47]. A different role has been assigned to keratin K17, whose expression is induced in epidermal keratinocytes upon environmental stress [48]. K17 appears to stimulate inflammatory responses by cytokine induction through signaling pathways and possibly even direct modulation of gene transcription [48,49,50,51].

Taken together, we posit that keratins have an overall protective function against microbial infection. This function is not limited to providing a structural barrier but includes active mechanisms that trigger complex responses.

## 3. Keratin-Microbe Interactions in Stratified Epithelia

In the following paragraphs, we will focus on specific host-pathogen interactions that have been described for stratified epithelia. Table 1 lists examples of pathogen-keratin interaction that utilize and disrupt the keratin-dependent barrier.

### 3.1. Epithelial Colonization

The commensal *Staphylococcus aureus* permanently colonizes the anterior part of the nasal cavity. The staphylococcal surface receptor clumping factor B (ClfB) plays a pivotal role in this process. It was shown that ClfB binds to epidermal K10 [52,72,73], which is typically found in cornified stratified epithelia [74,75]. The interaction between ClfB and K10 enhanced adherence of *Staphylococcus aureus* to epithelial cells and thereby supported epithelial colonization, notably in the squamous epithelial cells of the nasal epithelium [76]. In yeast two-hybrid binding assays ClfB also interacted with K8, which is predominantly expressed in simple epithelia [77], the physiological relevance of which remains to be assessed.

In another study, binding was detected between K4 and the surface serine-rich repeat protein Srr-1 of *Streptococcus agalactiae*, a commensal bacterium of the human gastrointestinal and female vaginal tract [53]. K4 is prominent in non-cornified stratified epithelia lining the oral mucosa, esophagus, and vagina [74,75]. Binding of Srr-1 was localized to the carboxyterminal 255 amino acids of K4 and was shown to be needed for adherence of *Streptococcus agalactiae* to epithelial cells in a dose-dependent fashion [53].

Interactions between keratins and bacterial surface proteins may be a rather wide-spread and common phenomenon as suggested by Tamura and Nittayajarn [78]. These authors found that soluble K8 bound to all of six group B streptococci strains that they tested as well as to four other gram-positive cocci, i.e., *Staphylococcus aureus*, *Lactococcus lactis*, *Enterococcus faecalis*, and *Streptococcus pyogenes*. An unresolved conundrum is whether keratins are exposed at the cell surface physiologically or need to be set free from their cytoplasmic compartment, for example by bacterial proteases.

### 3.2. Keratin Network Disruption

A very well examined situation of pathogen-keratin interaction in stratified epithelia is human papilloma virus (HPV) type 16 infection. During the infection cycle the viral E1^E4 protein accumulates in the upper layers of infected epithelia such as the stratified cervical epithelium comprising up to 30% of total cell protein in some lesions [54,79]. The E1^E4 protein forms amyloid-like fibers after cleavage of its aminoterminal 17 amino acids by the cytoplasmic cysteine protease calpain [80]. E1^E4 protein-containing amyloid fibers are initially detected in the suprabasal cells, which contain active calpain [80]. The multimeric E1^E4 protein-derived aggregates associate with keratin filaments containing K10, K13, K14, and K18 [54]. Based on co-localization, co-immunoprecipitation, and in vitro binding assays a direct interaction was described for K18 and the aminoterminus of E1^E4 protein [81]. Of note, keratin network dynamics were reduced in the presence of E1^E4 protein in cultured SiHa cervical epithelial cells [81]. The interaction between E1^E4 protein and keratin was favored by phosphorylation of T57 in E1^E4 protein through extracellular signal regulated kinase ERK [82]. In addition, other kinases such as cyclin-dependent kinase, protein kinase A and protein kinase Cα may be implicated [30,82]. The importance of E1^E4 protein activity for keratin network modification was further underscored by the exclusive detection of T57-phosphorylation of E1^E4 protein in the intermediate cell layers of epidermal raft cultures, i.e., within the cell layers in which productive infection occurs [82]. Furthermore, keratins become hyperphosphorylated and are ubiquitinylated in HPV-infected cells [54]. Remarkably, microtubules and actin filaments as well as the nuclear lamin IFs were unaffected [79]. A likely consequence of the selective keratin network collapse is enhanced release of viral particles which are then available for further infection of epithelial cells.

Herpes simplex virus type 2 (HSV-2) infects preferentially the skin and genital mucous membranes. It synthesizes the cytoplasmic ubiquitin-interacting US2 protein during the late phase of infection [83]. US2 has been implicated in the release of viral particles [84]. K18 was identified as a binding partner of US2 in a yeast two-hybrid screen [55]. This interaction was confirmed by co-immunoprecipitation experiments and co-localization studies of infected cultured cells. The keratin network in these cells was considerably altered presenting thickened and clumped keratin filaments, preferentially in the cell periphery. Later on, Murata et al. [56] observed that expression of the HSV-2 protein kinase US3, which is involved in cell morphology alterations and disruption of the actin cytoskeleton [85], enhanced K17 phosphorylation and ubiquitination. This was also accompanied by appearance of thicker keratin filaments and keratin network disruption. These authors [56] further presented evidence that the US3 kinase directly phosphorylated K17 and showed that this interaction elicits distinct cytopathic effects.

### 3.3. Induction of Inflammation

*Porphyromonas gingivalis* is a major etiological bacterium of periodontal disease. It secretes the lysine-specific protease gingipain [86,87]. Among multiple targets, keratins were described as potential substrates for this protease by Tancharoen and co-workers [57]. They detected a novel K6 fragment in the gingival crevicular fluid of periodontal disease patients. This 19 amino acid-long fragment was shown to be generated by lysine-specific gingipain treatment of cultured cells [57]. Interestingly, this peptide induced gingival fibroblast migration, secretion of interleukins 6 and 8, and production of monocyte chemoattractant protein 1 [57]. This example illustrates nicely how a pathogen destroys the keratin-based barrier and simultaneously initiates an inflammatory response.

### 3.4. Bacteriotoxicity

An extracellular protective function of keratins was recently described for the cornea. Peptide fragments derived from the carboxyterminus of keratin K6a were identified in a crude extract from differentiated cultured corneal epithelial cells [58]. These peptides exhibited strong antibacterial activity against *Pseudomonas aeruginosa* and, more importantly, against the ocular pathogens *Staphylococcus aureus* and *Streptococcus pyogenes* [58]. Detailed analysis of an amphipathic 19-mer peptide rapidly killed cytotoxic *Pseudomonas aeruginosa* in either water or at physiological ionic conditions. This was mediated through specific binding to the bacterial cytoplasmic membrane causing subsequent leakage [58]. These observations may explain how the corneal epithelium is protected from microbes such as *Staphylococcus aureus* that are resistant to lysozymes in tear fluid but populate the upper airway ([88] and Figure 1B). Since the expression of K6a is not restricted to the cornea [74,75], it may have similar functions in other epithelia such as the epidermis and various mucosal surfaces.

## 4. Intermediate Filaments Mediating Barrier Function in Simple Epithelia

Keratin IFs in simple epithelia are mostly concentrated underneath the plasma membrane with different degrees of polarization and cytoplasmic localization [89,90,91]. The most extreme distribution has been reported for enterocytes, in which keratin IFs are subapically enriched in a dense filamentous network just below the actin-rich terminal web, which anchors the apical microvilli ([92,93,94] and Figure 1A). High resolution imaging of vital intestinal mucosa of knock-in mice producing K8-yellow fluorescent protein showed nicely the subapical concentration of keratins together with submembraneous localization at the lateral membrane domains but no detectable cytoplasmic fluorescence [89] in contrast to fixed tissue samples [89,90]. Whether this arrangement performs the same structural reinforcing role as the pan-cytoplasmic keratin network in stratified epithelia is not so clear [33]. It is generally accepted, however, that keratins also play a crucial role in the stress response of simple epithelia [95,96,97]. In accordance, increased susceptibility to toxic liver damage was observed in Krt18^−/−^ mice [33]. These mice deposit K8 aggregates in hepatocytes which share features with Mallory bodies that are observed in alcoholic liver cirrhosis. In contrast, loss of K8 or relative excess of K18 over K8 prevent Mallory body formation although increased sensitivity to toxic liver damage is still detectable in these situations. Accordingly, defects in K8 and K18 were described in human liver disorders [98,99,100] and were also reported in chronic pancreatitis [101] and inflammatory bowel disease [102], while others have reported no predisposition of K8/K18/K19 variants to pancreatitis and inflammatory bowel disease [103,104,105]. Although the identified mutations are not lethal, they appear to negatively affect epithelial resilience, predisposing affected patients to a breakdown of the epithelial barrier, especially in the presence of physiological stress such as osmotic challenges and mechanical strain and, even more so, in pathological stress situations such as microbial insults [27,102]. In support, colonic hyperplasia colitis and rectal prolapse are observed in K8^−/−^ mice [106]. This phenotype is characterized by a marked increase in TCRβ-positive and CD4-positive T cells infiltrating the *lamina propria* of the colon mucosa which is coupled to enhanced Th2 cytokine (IL-4, IL-5 and IL-13) production [107]. Consistent with this observation it was recently shown that K8/K18 are able to bind to the inflammasome to regulate the IL-22 inflammatory response through IL-18 and maintain the barrier function of the intestinal epithelium, while K8^−/−^ mice display increased inflammation, barrier defects, and tumorigenesis through inflammasome activation [108]. Antibiotic treatment markedly decreased colonic inflammation [107]. Later on, Habtezion, et al. [109] demonstrated differential regulation of genes involved in apoptosis in K8^+/+^ vs. K8^−/−^ murine colonocytes resulting in apoptosis resistance in a microflora-dependent manner. Even heterozygous K8^+/−^ animals presented longer colonic crypts but did not exhibit increased apoptosis and inflammation. Yet, they displayed higher sensitivity to dextran sulphate sodium in a colitis disease model [110].

It was further shown by different groups that simple epithelial keratins protect cells in vitro against TNFα- and Fas ligand-mediated apoptosis [111,112,113]. Interestingly, K8/K18 co-localize with the cytoplasmic domain of TNF receptor 2 and moderate TNF-induced JNK intracellular signaling and NFκB activation [112]. In inflammatory bowel disease it was observed that the induced inflammatory response, in turn, leads to the release of damaging oxidants such as H_2_O_2_, HOCl, and OH_2_ [114], which induce mucosal injury [115] and cause impaired epithelial barrier function by perturbing the actin and microtubule cytoskeleton [116]. Oxidative stress is also known to induce K8 homodimer formation, which efficiently prevents filament assembly [99,102]. Homodimer formation has also been observed upon oxidative stress in liver explants and cultured intestinal cells expressing K8 mutants that have been identified in patients with inflammatory bowel disease [99,102]. The reported massive decrease of K8, K18, K19, and vimentin in concert with reduced phosphorylation of K8 in the mucosa of inflammatory bowel disease patients can be taken as additional indication that inflammatory response triggers keratin dysfunction as part of a mutually enhancing vicious cycle [117]. Remarkably, K8 levels and phosphorylation are restored or even elevated in intestinal bowel disease patients with clinical and endoscopic remission [117].

Another interesting link between inflammatory cytokine production and keratins has been described by Wang and colleagues [118]. They showed that IL-6 induces upregulation of the mRNA and protein levels of K8 and K18 in colon adenocarcinoma-derived Caco-2 BBE cells. K8 and K18 localize in a reticular pattern to the subapical region of these cells upon IL-6 treatment coincident with a decrease of paracellular flux. This response was abolished by K8 silencing. Furthermore, administration of dextran sodium sulfate (DSS) significantly increased intestinal permeability in IL-6 knockout mice compared to the wildtype suggesting that IL-6 mediates intestinal barrier protection via K8/K18 overexpression [118]. On the other hand, high IL-6 levels are also known to perpetuate the inflammatory state and tissue destruction in inflammatory bowel diseases, in part through induction of Th17 cells [119,120].

Abnormalities in transport of ions, protein mistargeting, and diarrhea have been noted in the colon upon keratin depletion even before the development of inflammation [121]. A likely reason is that keratins fulfil a general scaffolding function for membrane proteins [122]. Very recently Asghar and co-workers [123] observed a complete loss of the apical localizing chloride transporter (DRA) explaining the strong diarrhea phenotype occurring in patients with inflammatory bowel disease. Furthermore, a direct connection between higher levels of microbiota-produced short chain fatty acids in stool and decreased levels of the monocarboxylate transporter 1 (MCT1) was shown in K8^−/−^ colon [124]. Even more, keratins have been shown to bind directly to membrane proteins such as polycystin-1 and CFTR [125,126,127].

## 5. Pathogens Interfering with Barrier Function in Simple Epithelia

In the following paragraphs, we briefly summarize observations of specific microbe-host interactions in simple epithelia that involve keratins.

### 5.1. Pathogen Docking

**Figure 2:**
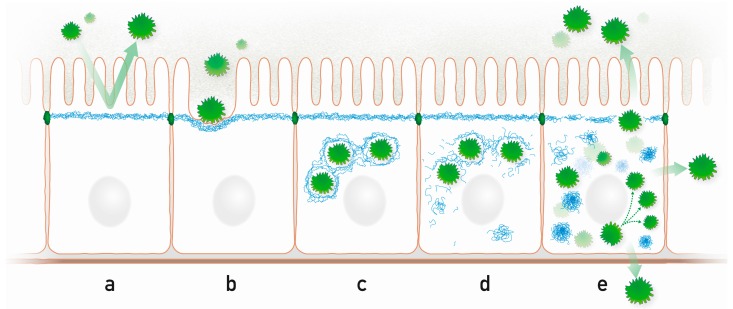
Highly schematic representation of processes that may occur during microbe-intermediate filament interaction in a simple epithelium. (**a**) The subapically enriched cytoplasmic IF system acts as an intracellular protective barrier; (**b**) IFs form together with the actin cytoskeleton pedestals for attached microbes; (**c**) Intracellular microbes are encaged by IFs; (**d**) Microbes disrupt the IF cage through kinase activities, which modify IF polypeptides and initiate the formation of cytoplasmic IF aggregates; (**e**) Released microbes proliferate and spread to neighboring cells and to the environment disrupting the protective apical IF network. Prominent cytoplasmic aggregates containing hyperphosphorylated IFs appear.

Enteropathogenic *Escherichia coli* (EPEC) are major pathogens causing severe gastroenteritis in humans [128] by causing attaching and effacing (A/E) lesions that are characterized by microvilli destruction [129] and subsequent disappearance of the terminal web [130]. Electron-dense zones are formed underneath the bacterial attachment sites that are rich in F-actin, myosin-II, villin, fodrin, and tubulin [130,131]. The attachment of EPEC was shown to involve the binding of the translocated intimin receptor (Tir) to the host adaptor protein Nck [132]. This interaction induced the recruitment of the neural Wiskott-Aldrich syndrome protein (N-WASP) and the actin-related protein (Arp2/3) complex which then led to the formation of an actin filament-rich pedestal. These studies were further extended by Batchelor and co-workers [59] showing that K18 is involved in this process. Evidence was presented that Tir interacts with K18, which in turn induced pedestal formation by actin accretion and cytoskeletal reorganization. In this way, a transmembrane bridge is formed connecting the cytoskeleton of the intestinal epithelial cell and the pathogen ([59] and Figure 2b).

Similarily, involvement of keratins as mediators for pathogen docking and uptake was also noted in intestinal *Salmonella enterica serovar typhimurium* infection [60,61]. This bacterium is responsible for worldwide epidemics because of increasing multi-drug resistance [133]. *Salmonella* pathogen invasion involves dramatic rearrangements of the host cytoskeleton and actin polymerization ([134,135,136,137,138] and Figure 2b). One of the essential proteins for invasion is the secreted invasion protein SipC [139]. Using a yeast two-hybrid system, Carlson and colleagues [60] identified an interaction between SipC and K18. Interestingly, expression of the dominant negative K18 mutant K18-R89C was shown to inhibit *Salmonella* invasion. It is also noteworthy that K18 is present in M-cells of the intestine [140] which are the major in vivo entry site of this pathogen [141]. Another interaction was described for K8 and the *Salmonella* type III secretion translocon protein SspC. The insertion of SspC into the host cytoplasm is required for *Salmonella* invasion and effector molecule translocation [61]. The type III secretion systems are used by more than 30 other bacterial pathogens, most notably by *Shigella* spp. [142], to establish infection though the delivery of effector proteins to the host cell. *Shigella flexneri* causes bacterial dysentery by invading colonic and rectal epithelium and causing severe mucosal inflammation and tissue damage resulting in abscesses and ulceration [143,144]. Very recently, Russo and co-workers [62] showed that K18 and vimentin interact with the carboxyterminus of the *Shigella* translocon pore protein IpaC. 

Taken together, these examples elucidate how pathogens use the mechanically stable keratin barrier as an anchor to bind to the cell membrane in order to fulfill host invasion (Figure 2b,c).

### 5.2. Induction of Cytotoxic Effects

Cytotoxic serine protease autotransporters of *Enterobacteriaceae* (SPATE) are implicated in cytotoxic effects. They promote their own secretion into the extracellular space through the type V secretion system [145] representing the most common mechanism used to release virulence factors by Gram-negative bacteria [146]. The class 1 SPATE Pet is a common toxin that was recently found to bind K8 [63]. It was further shown that the cytopathic effects of Pet are dependent on the presence/availability of K8. It was suggested that these effects were mediated through a keratin-dependent modulation of the clathrin-mediated endocytosis of Pet. 

### 5.3. Keratin Network Disruption

As described above for microbial infection of stratified epithelia, increased keratin phosphorylation was also observed in infected simple epithelia. Thus, Rotavirus infection, which is the most common cause of severe diarrhea in humans, was shown to increase hyperphosphorylated K8 [64]. This change was accompanied by reorganization and partial disruption of the keratin filament network without visible changes in the actin filament and microtubule networks (Figure 2d). At the same time, the soluble keratin pool was considerably increased. Subsequently, increased K8 and K18 phosphorylation has been shown to correlate with the progression of hepatitis B and C ([147,148] and Figure 2d,e). Another example of network disruption was described by Toivola et al. [149] using coxsackievirus B4 variants CVB4-V and CVB4-P, which induce acute/chronic pancreatitis and chronic pancreatitis, respectively. Infection with CVB4-V was shown to lead to an increase of mortality by 40% in K8^−/−^ mice compared to either wildtype or K18^−/−^ mice. Yet all animals displayed reorganization of the apicolateral K8/K18 network (Figure 2d,e) and loss of acini. The surviving K8^−/−^ mice also displayed enhanced signs of inflammation. In contrast, K8^−/−^ mice were less susceptible to CVB4-P infection compared to control animals and exhibited more efficient acinar repair. The network disruption observed during CVB4-P infection was shown to go along with phosphorylation of K8-S438 and K18-S35. Studies on keratin phosphorylation during CVB4-V infection have not been performed so far.

Another mechanism to disrupt the keratin network is proteolysis. This mechanism has been demonstrated for adenovirus. The adenovirus late-acting L3 23-kDa proteinase cleaves the aminoterminal head domain of K18 in different cell culture systems [65,66]. In conjunction with shut down of host translation this was shown to result in keratin network disruption with formation of cytoplasmic granular aggregates thereby favoring cell lysis and release of mature virus particles ([65,66] and Figure 2d,e). Similarly, it was reported that 2A proteinase cleaves K8 during a late stage of the infection cycle with human rhinovirus serotype 2 in HeLa cells [67]. This enzyme is also produced by other rhinoviruses and enteroviruses including coxsackievirus B4 [67].

Another example of microbial proteolytic effects is provided by the Gram-*negative Chlamydia trachomatis*, which is the leading cause of sexually transmitted bacterial disease worldwide. The intracellular pathogen proliferates in cytoplasmic vacuoles that are surrounded by a stabilizing dense coat of F-actin and IFs ([150] and Figure 2c). *Chlamydia trachomatis* secretes a protease that is referred to as chlamydial protease-like activity factor (CPAF) and was shown to cleave K8 and K18 [68,150]. This processing did not prevent keratin filament formation but presumably modified the structural scaffolding properties of the keratin filament network [150]. Similarly, CPAF of the related *Chlamydia* *pneumoniae* cleaves K8 and probably also K18 [69]. It is assumed that these proteolytic activities alter the cytoskeletal actin- and IF-based envelope of the vacuole to support vacuolar expansion and thereby enhance intravacuolar chlamydial replication ([151] and Figure 2c–e).

### 5.4. Pathogen Proliferation and Survival

Chagas’ disease is caused by the protozoan parasite *Trypanosoma cruzi*. The *Trypanosoma* glycoprotein gp85 has been implicated in cell invasion [152]. The gp85-derived nonapeptide TS9 has significant cell binding capacity and was found to bind keratins and vimentin in in vitro binding assays [70]. Using LLC-MK_2_ kidney epithelial cells, it was further demonstrated that *Trypanosoma cruzi* adhesion and cell infection can be reduced by TS9 peptide resulting in a reduced number of parasites per cell. 

Examination of the spore stage of the microsporidian nerve parasite *Spraguea lophii* suggested a mechanism by which a pathogen may utilize the keratin filament network for survival [71]. It was shown that these spores are stabilized by K4 and K13 on their outer envelope thereby preventing spore activation in Hepes-buffered conditions at pH 7.0. Changes to more basic conditions by adding polyanionic mucins led to increased keratin phosphorylation, which resulted in keratin dissociation and disassembly of the outer envelope followed by polar tube release. The polar tube pierces cell membranes to act as a conduit for the sporoplasm into a new host cell.

## 6. The *C. elegans* Intestine as a Model System for Investigating Intermediate Filament-Microbe Interactions

The striking arrangement of IFs in a dense fibrous layer just below the apical terminal web is conserved in vertebrates including fish [153], amphibians [154], and mammals [11] as well as in the nematode *Caenorhabditis elegans*, where IFs localize to the prominent electron-dense endotube [155,156,157]. Using transgene strains expressing a fluorescent IF reporter [158] in a mutagenesis screen, the intestinal filament organizer IFO-1 was identified [159]. It acts as a structural component to localize the IF-rich endotube to the periluminal subapical region of intestinal cells [159]. Animals lacking IFO-1 showed a complete loss of the endotube and a dilated lumen. They presented severe growth and development defects, which are most likely caused by impaired nutrient uptake [our unpublished results and 159]. We propose that these and other recently characterized mutants may exhibit increased sensitivity against microbial and toxic insults. This notion is supported by the identification of IFO-1 as a bacterial pore forming toxin-regulated target of MAP kinase [160]. Furthermore, similar alterations of the endotube concurrent with cytoplasmic invaginations and luminal dilation have been described in the intestine of worms that were infected with the microsporidian parasite *Nematocida parisii* [161]. The infection caused rearrangements of the apically restricted actin and IF cytoskeleton, resulting in gaps which are presumably used for the non-lytic exit of intracellularly synthesized spores. Similar luminal alterations were observed by Stutz and colleagues [162] using fungal *Coprinopsis cinerea* lectin 2 (CCL2), a non-immunoglobulin carbohydrate-binding protein without enzymatic activity. This phenotype resulted in developmental delay and premature death. Ultrastructural analyses revealed highly damaged intestinal cells with loss of microvilli, actin depolymerization, and striking invaginations of the apical plasma membrane [162]. 

## 7. Conclusions and Outlook

Given the crucial function of the keratin cytoskeleton as a barrier to protect the body from environmental insults, microbes have developed ingenious mechanisms to break down this barrier for local colonization and subsequent proliferation and spreading. Strengthening the keratin-based barrier may therefore protect the organism from microbial challenges. In agreement, it has been shown that the probiotic bacterial strain *Bifidobacterium breve* increases the expression of K8 in infected colon carcinoma-derived HT29 cells [163]. A mechanistic understanding of the manifold molecular interactions between microbes and the epithelial IF cytoskeleton is still in its infancy. Figure 2 illustrates some of the processes that may occur during microbe-intermediate filament interaction in a simple epithelium. Key questions that need to be addressed in the future are: (1) What are the precise structural and functional consequences of microbe-induced post-translational IF modifications? (2) How do the post-translational IF modifications affect microbial infection and propagation? (3) How do the unique mechanical properties of the IF system affect microbe-host interaction? (4) How do microbe-induced IF alterations affect the innate immune response? (5) How are IFs interlaced in microbe-related tumorigenesis (see, for example [164])?

The elucidation of these mechanisms will help to increase our functional understanding of the keratin cytoskeleton and will provide novel strategies for interfering with microbial infections.

## Figures and Tables

**Table 1:** List of specific pathogen-keratin interactions in stratified (orange) and simple (green) epithelia.

	Pathogen	Mechanism	Effect	Cell Type	Reference
	*Staphylococcus aureus*	Staphylococcal surface protein clumping factor B (ClfB)-dependent adherence to K10	Epithelial colonization	Squamous nasal epithelial cells	[52]
	*Streptococcus agalactiae*	Streptococcal surface-localized serine-rich repeat protein Srr-1 binding to K4	Epithelial colonization	Saliva extracts	[53]
	Human papilloma virus type 16	Association of HPV type 16 E1^E4 protein with K18 followed by K18-S33 and K18-S52 phosphorylation and ubiquitinylation	Keratin network disruption	SiHa and HaCaT cells	[54]
	*Herpes simplex* virus type 2	Association of US2 with K18	Keratin network disruption	Vero and A431 cells	[55]
	*Herpes simplex* virus type 2	Association of US3 with K17 followed by keratin phosphorylation and ubiquitinylation	Keratin network disruption	Hep2 cells	[56]
	*Porphyromonas gingivalis*	Cleavage of K6 at K357-Y358 and K378-Q379 by lysine-specific gingipain	Induction of inflammation	Gingival epithelial cells	[57]
	*Pseudomonas aeruginosa*	Release of K6-derived antibacterial peptides	Bacteriotoxicity	hTCEpi cells	[58]
	Enteropathogenic *Escherichia coli*	K18-dependent actin filament reorganization	Pathogen docking	HeLa cells	[59]
	*Salmonella enterica serovar typhimurium*	Interaction of secreted *Salmonella* invasion protein SipC with K18	Pathogen docking	HEp-2 cells	[60]
	*Salmonella enterica serovar typhimurium*	Interaction of *Salmonella* type III secretion translocon protein SspC with K8	Pathogen docking	HeLa cells	[61]
	*Shigella flexneri*	Binding of *Shigella* translocon pore protein IpaC to K18	Pathogen docking		[62]
	*Enterobacteriaceae*	Binding of the serine protease autotransporter of *Enterobacteriaceae* Pet to K8	Induction of cytotoxicity	HT-29 and HEp-2 cells	[63]
	Rotavirus	Phosphorylation of K8	Keratin network disruption	HT29 cells	[64]
	Adenovirus	Cleavage of aminoterminal K18 head domain at position 73	Keratin network disruption	HeLa and 293 cells	[65,66]
	Rhinovirus	Cleavage of aminoterminal K8 head domain at position 14 by 2A proteinase	Keratin network disruption	HeLa cell extracts	[67]
	*Chlamydia trachomatis*	Cleavage of K8 by chlamydial protease-like activity factor CPAF	Keratin network disruption	HeLa cells	[68]
	*Chlamydia pneumoniae*	Cleavage of K8 and K18 by chlamydial protease-like activity factor CPAF	Keratin network disruption	HL cells	[69]
	*Trypanosoma cruzi*	Binding of peptide TS9 of glycoprotein gp85 to K8/K18 (K14, K19, K20)	Cytoplasmic proliferation	LLC-MK_2_ cell extract	[70]
	*Spraguea lophii*	Phosphorylation of K4 and K13 in the outer spore envelope	Polar tube release		[71]
